# Biochar production under different pyrolysis temperatures with different types of agricultural wastes

**DOI:** 10.1038/s41598-024-52336-5

**Published:** 2024-02-01

**Authors:** El-Sayed Khater, Adel Bahnasawy, Ramy Hamouda, Amr Sabahy, Wael Abbas, Osama M. Morsy

**Affiliations:** 1 Agricultural and Biosystems Engineering Department, Faculty of Agriculture, Benha University, P.O. Box 13736, Moshtohor, Toukh, Kalubia Egypt; 2 Faculty of Organic Agriculture, Heliopolis University, P.O. Box 11785, Cairo, Egypt; 3 Institute of Agricultural Engineering Research, Agriculture Research Center, Doki, Giza, Egypt; 4 Basic and Applied Science Department, College of Engineering and Technology, Arab Academy for Science and Technology and Maritime Transport (AASTMT), P.O. Box 2033, Cairo, Egypt

**Keywords:** Ecology, Environmental sciences, Engineering

## Abstract

The main aim of this study is to determine the physical and chemical properties of biochar synthesized from different materials (straw rice, sawdust, sugar cane, and tree leaves) at different pyrolysis temperatures (400, 600, and 800 °C). The physical and chemical properties such as moisture content, water holding capacity, bulk density, and porosity; and pH, electrical conductivity (EC), organic matter, organic carbon, total nitrogen, potassium, phosphorus, calcium, magnesium, sodium, and sulfur were determined, respectively. The results show that the biochar yield decreased with increasing pyrolysis temperature, and the values of the analyzed properties varied depending on the type of biochar and pyrolysis temperature. The moisture content ranged from 1.11 to 4.18%, and the water holding capacity ranged from 12.9 to 27.6 g water g^−1^ dry sample. The highest value of bulk density (211.9 kg m^−3^) was obtained from sawdust at a pyrolysis temperature of 800 °C. The porosity values ranged from 45.9 to 63.7%. The highest values of pH and EC (10.4 and 3.46 dS m^−1^) were obtained from tree leaves at a pyrolysis temperature of 800 °C. Total organic matter ranged from 66.0 to 98.1%, total organic carbon ranged from 38.3 to 56.9%, and total nitrogen ranged from 0.4 to 1.9%. The highest values of phosphorus and calcium content (134.6 and 649.0 mg kg^−1^) were obtained from sugar cane at a pyrolysis temperature of 800 °C. The magnesium, sodium and sulfur content had ranges of 10.9–51.7, 1124–1703 and 3568–12,060 mg kg^−1^, respectively.

## Introduction

Biochar is a carbon-rich material which is produced by of heating biomass such as wood, manure, or leaves, at temperatures of 400–700 °C in a closed container in which oxygen is absent or depleted^1^. Biochar is used as an amendment to soil growth media and is produced by the pyrolysis of organic wastes. The pyrolysis process produces biochar and two additional materials—syngas and bio-oil—that have commercial value as energy sources. Recently, biochar has received considerable attention as a soil amendment for increasing agricultural productivity^2,3^. Alghashm *et al*^4^ mentioned that the pyrolysis temperature for preparing biochar ranged from 400 to 900 °C. Jindo *et al*^5^ selected some organic wastes (rice husk, rice straw, wood chips of apple tree and oak tree) with different characteristics and were pyrolyzed at different temperatures (400, 500, 600, 700 and 800 °C) in order to optimize the physicochemical properties of biochar as a soil amendment.

Scientists and policymakers are beginning to recognize the important role of biochar in the reduction of greenhouse gas emissions, production of renewable energy, mitigation of waste, and application as a soil amendment^6^. Biochar is used in many fields. In agriculture, it is used as an organic fertilizer that degrades over a long time. When added to soil, it positively affects the soil fertility, the total biogenic components, physical and water characteristics, and biological features^7,8^.

The physical characteristics of biochar render it a useful tool for environmental management. The physical properties of biochar can affect soil systems directly and indirectly. Different soils have distinct physical features that are dependent upon the nature and relative quantity of their mineral and organic matter content, as well as the association between the minerals and organic matter^9^. Biochar added to a soil mixture can significantly contribute to the physical nature of the system by affecting the penetration depth, structure, texture, porosity, and consistency by changing the surface area, particle size distribution, pore size distribution, density, and packing^10^. The influence of biochar on the physical features of soil can then have a direct impact on plant growth because the penetration depth and availability of air and water in the root zone are largely determined by the physical make-up of the soil horizons. Biochar’s presence in soil directly affect the soil’s response to water, as well as its aggregation, workability during soil preparation, swelling shrinking dynamics, permeability, capacity to retain cations, and response to ambient temperature changes. In addition, many chemical and biological aspects of soil fertility can be indirectly inferred from these physical properties, such as the physical availability of sites for chemical reactions and the provision of protective habitats for soil microbes^11,12^.

Biochar has higher nutrient retention capacity and higher resistance to degradation by microorganisms which are due to chemical and colloidal structure^13^. Despite of high significant losses of nutrients during pyrolysis, biochar has positive responses on the soil, because it neutralizes the toxins and improves the physical properties of soils such as water retention as well as develop a protection against heavy metal pollution and reduce soil compaction^11^. Physical and chemical features of biochar vary by the variation of the properties of raw materials used and conditions of pyrolysis^14^.

Soil properties such as physical and biological properties could be improved by using biochar and it is used as preventive against heavy metal stress of soil^15,16^. Biochar produced from cotton stalk has capacity of holding higher Cadium (Cd) in soil contaminated with Cd. It reduced the biological effect of Cd in soil by changing the morphological structure of Cd^17^. Several studies have been covered the production of biochar from agriculture wastes which was used to prevent the heavy metals and organic pollutants in soil and water^18^.

Biochar could be used in improving anaerobic digestion as mentioned by Valentin *et al*^19^ whose stated that properties such as specific surface area (SSA), cation exchange capacity, presence of functional groups and electrical conductivity were found favorable for increased methane production, reduction of lag phase, and adsorption of inhibitors.

The published works on biochar application to soil have predominantly focused on agronomic benefits, while the physical and chemical properties of the produced biochar and their effects on soil structure and texture have received little attention. Therefore, there is a lack of information about aspects of biochar that are important for plant growth and soil improvement. Thus, the main aim of this work is to determine same properties of biochar. In particular, the physical properties (bulk density, moisture content, water holding capacity, and porosity) and chemical properties (pH, Electrical Conductivity (EC), organic matter, organic carbon, total nitrogen, and potassium) of different types of biochar were analyzed at different pyrolysis temperatures.

## Materials and methods

The experiments were carried out at the Agricultural and Bio-Systems Engineering Department, Faculty of Agriculture, Moshtohor, Benha University, during the months of April and May, 2023 season under the regulations of International, National and Benha University which are consistent with the national and international guidelines and legislation. Biochar was produced from certain agricultural wastes, manly straw rice, sawdust, sugar cane plant residues, and tree leaves. The physical and chemical properties that are relevant to the manufacturing of biochar using these four materials are listed in Tables 1 and 2, respectively.

**Table 1:** Physical properties of raw materials used in the production of biochar.

Properties	Raw materials			
	Straw rice	Sawdust	Sugar cane	Tree leaves
Moisture content (%)	11.6^a^	8.7^a^	36.2^c^	22.7^b^
Water holding capacity (g water g^−1^ dry)	1.4^a^	1.9^b^	3.3^b^	4.6^c^
Bulk density (kg m^−3^)	180^a^	230^b^	426^d^	276^c^
Porosity (%)	43.50^a^	66.30^c^	69.96^c^	54.60^b^

Means on the same row with different superscripts are significantly different (*p* < 0.05).

**Table 2:** Chemical properties of raw materials used in the production of biochar.

Properties	Raw materials			
	Straw Rice	Sawdust	Sugar Cane	Tree Leaves
pH	6.8^b^	6.3^a^	7.10^b^	7.6^c^
Electrical conductivity, EC (dS m^−1^)	1.2^a^	1.13^a^	3.10^c^	1.75^b^
Total organic matter (%)	77.06^b^	82.13^b^	34.48^a^	92.4^c^
Total organic carbon (%)	44.7^b^	47.64^b^	20.00^a^	53.60^c^
Total nitrogen (%)	0.49^a^	0.62^a^	1.62^b^	3.2^c^
Total phosphorus (%)	0.32^a^	1.3^b^	1.12^b^	1.8^c^
Total potassium (%)	0.53^a^	0.89^b^	2.36^b^	2.9^b^
Total calcium (%)	1.13^a^	2.3^a^	2.8^ab^	3.11^c^
Total magnesium (%)	0.74^a^	0.4^a^	1.03^ab^	1.14^b^
Total sodium (%)	0.14^a^	0.19^b^	0.21^b^	0.13^a^
Total sulfur (%)	2.14^a^	3.11^b^	4.13^c^	2.4^a^
C/N ratio	91.22:1^c^	76.84:1^b^	12.35:1^a^	16.75:1^a^

Means on the same row with different superscripts are significantly different (*p* < 0.05).

All study materials (straw rice, sawdust, sugar cane plant residues, and tree leaves) were sun-dried and cut into small pieces (less than 4–5 cm), which were then inserted into a ceramic vessel (500 cm^3^) and placed in a commercial electric furnace (SOMO-01 Isuzu, Japan). The material was charred for 6 h at different pyrolysis temperatures (400, 600, and 800 °C). The experiments were repeated 3 times and averages were taken.

### Biochar yield

The biochar yield was calculated using Eq. (1):

1 $$biochar\;{\text{yield}} = \frac{{weight\;of\;pyrolysis\;materials\;{\text{(g)}}}}{{mass\;of\;raw\;material\;input\;{\text{(kg)}}}}$$

### Biochar physical properties

Moisture content (MC) of biochar was determined by drying the product at 105 °C for 24 h or to a constant weight according to ASAE^20^.

The Water Holding Capacity (WHC) of biochar was determined by measuring the weight of a wet sample (W_*i*_). This could be done by placing samples in a beaker for 1–2 days using distilled water. Excess water was drained through Whatman #2 filter paper, and the saturated sample was weighed again (*W*_*s*_). The water holding capacity was calculated using the following equation (Ahn *et al*^21^):

2 $$WHC = \frac{{\left\{ {\left( {W_{s} - W_{i} } \right) + MC \times W_{i} } \right\}}}{{\left\{ {\left( {1 - MC} \right) \times W_{i} } \right\}}}$$

where *W*_*i*_ is the initial weight of the biochar (g), *W*_*s*_ is the saturated weight of the biochar (g), and *MC* is the initial moisture content of the sample (decimal).

The bulk density (BD) of biochar was determined by placing the biochar in a known volume scaled flask (1 L) and the mass of biochar in the scaled flask was measured. The sample weight was recorded and the bulk volume was measured. The bulk density was calculated using Eq. (3):

3 $$BD = \frac{{mass\;{\text{of}}\;{\text{biochar}}}}{{Bulk\;{\text{volume}}\;{\text{of}}\;{\text{biochar}}}} \times {100}$$

Biochar porosity (*ε*_*a*_) was calculated using the following equation from^22–24^:

4 $$\varepsilon_{a} = 1 - \rho_{wb} \left( {\frac{MC}{{\rho_{w}^{{}} }} + \frac{DM \cdot OM}{{\rho_{om} }} + \frac{{DM \cdot \left( {1 - OM} \right)}}{{\rho_{ash} }}} \right) \, \times {100}$$

where *ε*_*a*_ is the biochar porosity (%), *ρ*_*w*_ is the water density (kg m^−3^), *ρ*_*wb*_ is the biochar bulk density (kg m^−3^), ρ_ash_ is the ash density (kg m^−3^), *ρ*_*om*_ is the organic matter density (kg m^−3^), DM is the dry matter (decimal), and OM is the organic matter (decimal).

### Biochar chemical properties

Electrical conductivity and pH were measured in a 1:5 (*v*/*v*) material/water extract using a glass electrode. Organic carbon (OC) was determined by using the dry combustion method at 540 °C for 4 h, as specified by^25^, which involves heating a biochar sample in an oxygen-free environment until it loses all its volatile matter, then weighing the residue and calculating the organic carbon content by subtracting the inorganic carbon content. Organic matter was measured by combustion at 550 °C for 8 h according to^26^, and total nitrogen (TN) was measured by Kjeldahl digestion (model VAPODEST; range 0.1 mg to 200 g N; Germany)^27^. Potassium (K) content was determined by atomic absorption (model EMI9783B; range of 190–930 nm; USA), and phosphorus (P) content was determined using the calorimetric method^28^. The quantities of calcium (Ca), magnesium (Mg), and sodium (Na) were determined by a flame photometer (model Jenway PFP7; range0–160 mmol L^−1^; USA). Sulfur content was determined by using barium chloride following^29^.

### Statistical analysis

The data were subjected to analysis using statistical package SPSS version 21 in which one way ANOVA and Duncan Multiple Range Test (DMRT) were performed at significance level of (*p* < 0.05) at 95% confidence limit to know the significant differences between the treatment means for different parameters.

## Results and discussion

### Biochar yield

**Figure 1:**
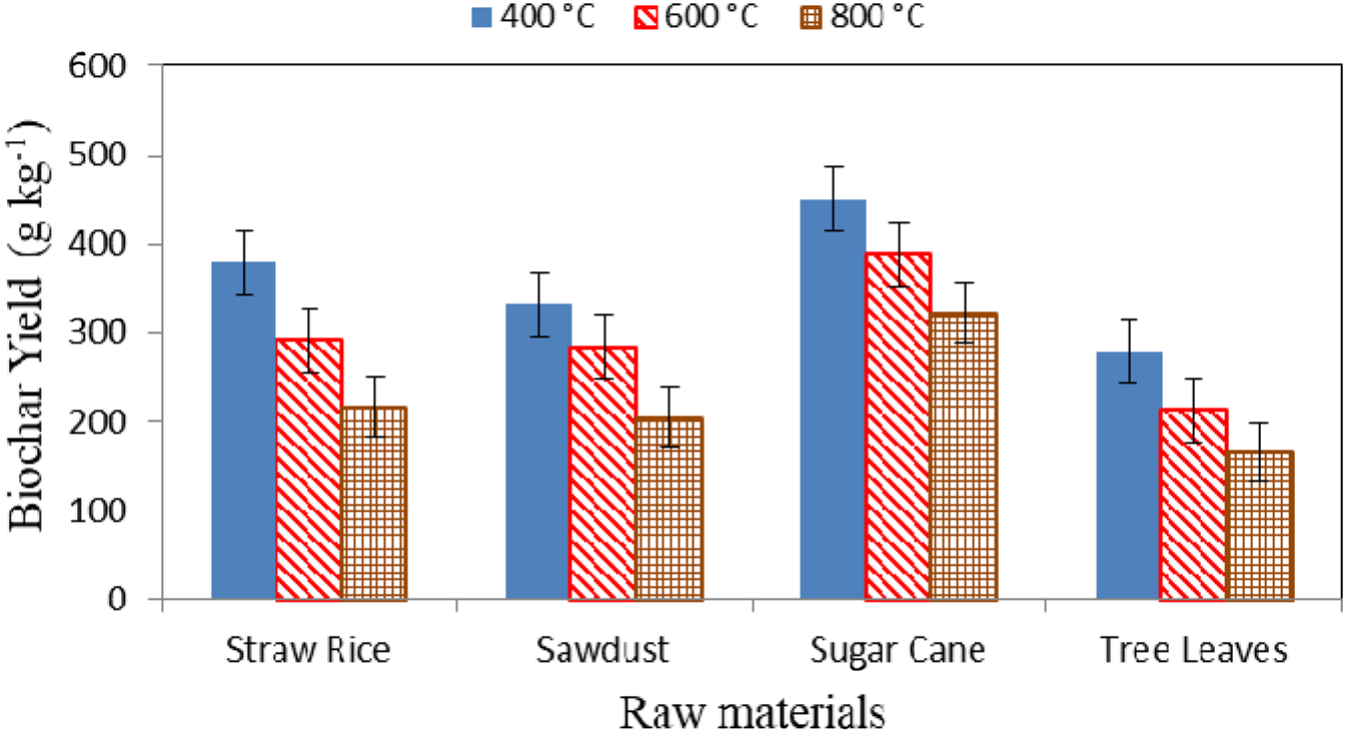
Biochar yield for different materials from 6 h of pyrolysis at different temperatures.

Table 3 and Fig. 1 show the biochar yield for different biochar types (straw rice, sawdust, sugar cane, and tree leaves) at different pyrolysis temperatures (400, 600, and 800 °C). The results indicate that the biochar yield decreased with increasing pyrolysis temperature. Increasing the temperature from 400 to 800 °C the biochar yield significantly decreased from 378.2 to 216.7 g kg^−1^ (57.29% decrease), 331.4 g kg^−1^ to 204.1 g kg^−1^ (61.59% decrease), 450.1 g kg^−1^ to 322.5 g kg^−1^ (71.65% decrease), and 277.9 g kg^−1^ to 165.0 g kg^−1^ (59.37% decrease) for straw rice, sawdust, sugar cane plant residues, and tree leaves, respectively. The biochar yield decreased with increasing pyrolysis temperature as a result of the increased burning rate, because the variation of lignin and cellulose content of biomass and conversion of organic matter to ash, which reduced the carbon content of the biochar. These results agree with those obtained by Jindo *et al*^5^. They found that the yield of biochar from apple tree branch, tree oak, rice husk, and rice straw decreased from 283 to 155 g kg^−1^, 358 to 191 g kg^−1^, 486 to 320 g kg^−1^, and 393 to 183 g kg^−1^, respectively, when the pyrolysis temperature increased from 400 to 800 °C.

**Table 3:** Biochar yield for different materials from 6 h of pyrolysis at different temperatures.

Temperature (°C)	Biochar types			
	Straw rice	Sawdust	Sugar cane	Tree leaves
400	378.2^h^	331.4^g^	450.1^j^	277.9^d^
600	291.3^e^	284.2^d^	389.6^i^	213.3^c^
800	216.7^c^	204.1^b^	322.5^f^	165.0^a^

Means with different superscripts are significantly different (*p* < 0.05).

The results also indicate that the highest biochar yield (450.1 g kg^−1^) was obtained from sugar cane at a pyrolysis temperature of 400 °C, because the sugar cane content higher total solids compared to the other materials used in this study. While the lowest biochar yield (165.0 g kg^-1^) was obtained from tree leaves at a pyrolysis temperature of 800 °C. These results agree with those obtained by Sarfraz *et al*^30^. They found the highest value of biochar yield was obtained at pyrolysis temperature of 400 °C, while, the lowest value of biochar yield was obtained at pyrolysis temperature of 700 °C.

### Physical properties

Table 4 shows the physical properties (moisture content, water holding capacity, bulk density, and porosity) of the different types of biochar (straw rice, sawdust, sugar cane, and tree leaves) at different pyrolysis temperatures (400, 600, and 800 °C). The results indicate that the moisture content (MC) decreased with increasing pyrolysis temperature. Increasing the pyrolysis temperature from 400 to 800 °C significantly decreased the MC from 2.64 to 1.11%, 2.59 to 1.34%, 3.17 to 1.66%, and 4.18% to 2.19% for straw rice, sawdust, sugar cane, and tree leaves, respectively. The results also show that the highest moisture content (4.18%) was obtained from tree leaves at a pyrolysis temperature of 400 °C, while the lowest moisture content (1.11%) was obtained from straw rice at a pyrolysis temperature of 800 °C.

**Table 4:** Physical properties of different biochar types.

Biochar types	Temperature (°C)	Physical properties			
		MC (%)	WHC* (g Water/g dry sample)	BD (kg m^−3^)	Porosity (%)
Straw rice	400	2.64^h^	12.9^a^	161.5^a^	63.7^g^
	600	1.29^b^	15.7^b^	170.6^b^	60.4^f^
	800	1.11^a^	22.5^c^	187.1^c^	56.1^d^
Sawdust	400	2.59^h^	20.3^c^	195.0^d^	51.0^c^
	600	1.50^c^	21.2^c^	205.5^f^	47.2^a^
	800	1.34^b^	24.1^d^	211.9^g^	45.9^a^
Sugar cane	400	3.17^i^	24.9^e^	175.7^b^	61.8^f^
	600	1.94^e^	26.5^e^	186.2^c^	56.9^de^
	800	1.66^d^	27.6^ef^	194.1^d^	54.3^d^
Tree leaves	400	4.18^j^	20.8^c^	188.0^c^	55.5^d^
	600	2.41^g^	21.5^c^	192.7^cd^	52.5^c^
	800	2.19^f^	24.8^de^	199.4^de^	49.2^b^

Means on the same column with different superscripts are significantly different (*p* < 0.05).

*WHC is water holding capacity.

The water holding capacity (WHC) significantly increased with increasing pyrolysis temperature. Increasing the pyrolysis from 400 to 800 °C increased the water holding capacity from 12.9 to 22.5, 20.3 to 24.1, 24.9 to 27.6, and 20.8 to 24.8 g water g^−1^ dry for straw rice, sawdust, sugar cane, and tree leaves, respectively. The highest WHC (27.6 g water g^−1^ dry) was obtained from tree leaves at a pyrolysis temperature of 800 °C, while the lowest WHC (12.9 g water g^−1^ dry) was obtained from straw rice at a pyrolysis temperature of 400 °C. These results agreed with those obtained by Alkhasha *et al*^31^. They found that the WHC was increased with increasing pyrolysis temperature for date palm wastes.

The bulk density (BD) also significantly increased with increasing pyrolysis temperature. Increasing the pyrolysis temperature from 400 to 800 °C increased the bulk density from 161.5 to 187.1, 195.0 to 211.9, 175.7 to 194.1, and 188.0 to 199.4 kg m^−3^ for straw rice, sawdust, sugar cane, and tree leaves, respectively. The highest bulk density (211.9 kg m^−3^) was obtained from sawdust at a pyrolysis temperature of 800 °C, while the lowest bulk density (161.5 kg m^−3^) was obtained from straw rice at a pyrolysis temperature of 400 °C.

The porosity decreased with significantly increasing pyrolysis temperature. Increasing the pyrolysis temperature from 400 to 800 °C decreased the porosity from 63.7 to 56.1%, 51.0 to 45.9%, 61.8 to 54.3%, and 55.5 to 49.2% for straw rice, sawdust, sugar cane, and tree leaves, respectively. The highest porosity (63.7%) was obtained from straw rice at a pyrolysis temperature of 400 °C, while the lowest porosity (47.2%) was obtained from sawdust at a pyrolysis temperature of 800 °C.

The biochar porosity was dependent on the bulk density and moisture content of biochar, and the porosity decreased with increasing bulk density and moisture content. The results indicate that the porosity of biochar decreased from 63.7 to 56.1%, 51.0 to 45.9%, 61.8 to 54.3%, and 55.5 to 49.2% for straw rice, sawdust, sugar cane, and tree leaves, respectively, when the bulk density increased from 161.5 to 187.1, 195.0 to 211.9, 175.7 to 194.1, and 188.0 to 199.4 kg m^−3^ and the moisture content increased from 2.64 to 1.11%, 2.59 to 1.34%, 3.17 to 1.66%, and 4.18 to 2.19%. These results agree with those obtained by Brewer *et al*^32^. They found the biochar density increased from 250 to 600 kg m^-3^, when the pyrolysis temperature increased from 350 to 450 °C for wood biochars.

### Biochar chemical properties

Table 5 shows the analyzed chemical characteristics (pH, EC, organic matter, organic carbon, total nitrogen, potassium, phosphorus, calcium, magnesium, sodium, and sulfur content) of the different types of biochar (straw rice, sawdust, sugar cane, and tree leaves) at different pyrolysis temperatures (400, 600, and 800 °C).

**Table 5:** Chemical properties of different biochar types.

Biochar types	Temperature (°C)	Chemical properties										
		pH	EC (dS m^−1^)	OM* (%)	OC** (%)	TN*** (%)	K**** (%)	P (mg kg^−1^)	Ca (mg kg^−1^)	Mg (mg kg^−1^)	Na (mg kg^−1^)	So_4_ (mg kg^−1^)
Straw rice	400	8.2^d^	1.48^d^	66.0^a^	38.3^a^	0.9^c^	0.6^a^	47.6^a^	241.3^a^	10.9^a^	1124^b^	3568^a^
	600	8.3^d^	2.07^e^	85.9^d^	49.8^d^	0.7^b^	1.3^b^	58.6^b^	254.9^b^	11.2^a^	1231^d^	4235^b^
	800	9.4f.	2.90f.	90.5^de^	52.5^de^	0.4^a^	1.6^c^	62.3^b^	264.2^b^	13.2^b^	1329^e^	4360^c^
Sawdust	400	7.3^b^	0.94^a^	74.8^c^	43.4^c^	1.4^ef^	1.3^b^	65.5^bc^	491.6^ g^	21.4^c^	1034^a^	9752^d^
	600	7.5^b^	1.16^b^	94.5^e^	54.8f.	1.0^c^	2.5f.	106.2f.	546.1^i^	25.5^d^	1046^a^	9854^d^
	800	7.6^bc^	1.52^d^	96.7f.	56.1f.	0.5^a^	2.7^ fg^	121.3^ h^	573.3^j^	27.8^e^	1109^b^	10138^e^
Sugar Cane	400	6.6^a^	0.82^a^	87.8^d^	50.9^d^	1.9^ g^	2.2^e^	77.3^e^	513.1^ h^	47.2^i^	1604^ g^	11235^ h^
	600	7.5^b^	1.03^ab^	97.2f.	56.4f.	1.8^ g^	3.1^i^	117.0^ g^	621.5^ k^	48.4^i^	1624^ g^	11561^i^
	800	8.9^e^	1.27^bc^	98.1f.	56.9^ fg^	1.3^e^	3.5^j^	134.6^i^	649.0^ l^	51.7^j^	1703^ h^	12060^j^
Tree Leaves	400	8.7^e^	2.11^e^	71.0^b^	41.2^b^	1.5f.	1.8^d^	59.6^b^	353.7^c^	30.7^e^	1204^c^	10334f.
	600	9.2f.	2.95f.	90.7^e^	52.6^e^	1.2^e^	2.6f.	67.1^c^	421.2^d^	33.1^e^	1324^e^	10609^ g^
	800	10.4^ g^	3.46^ g^	95.5^ef^	55.4f.	0.7^b^	2.9^ h^	70.9^d^	444.9f.	39.0^ h^	1509f.	11241^ h^

Means on the same column with different superscripts are significantly different (p < 0.05).

*OM, organic matter.

**OC, organic carbon.

***TN, total nitrogen.

****K, potassium.

The results indicate that the pH significantly increased with increasing pyrolysis temperature. Increasing the pyrolysis temperature from 400 to 800 °C increased the pH from 8.2 to 9.4, 7.3 to 7.6, 6.6 to 8.9, and 8.7 to 10.4 for straw rice, sawdust, sugar cane, and tree leaves, respectively. These results agree with those obtained by Alghashm *et al*^4^. They found the pH of biochar increased from 9.19 to 12.52 with pyrolysis temperature ranging from 400 to 900 °C. The results also indicate that the highest pH (10.4) was obtained from tree leaves at a pyrolysis temperature of 800 °C, while the lowest pH (6.6) was obtained from sugar cane at a pyrolysis temperature of 400 °C. However, biochar pH values may vary depending on the feedstock and production process. The observed increase in the pH of the four biochar types at higher temperatures is probably a consequence of the relative concentration of non-pyrolyzed inorganic elements that were present in the original feedstocks^33^. They found the pH was increased from 7.9 to 8.6, 5.9 to 7.2, 8.7 to 10.3 and 5.8 to 8.0, when the pyrolysis temperature increasing from 400 to 500 °C for peanut hull, pecan shell, poultry litter and switch grass, respectively.

The results also indicate that the EC significantly increased with increasing pyrolysis temperature. Increasing the pyrolysis temperature from 400 to 800 °C increased the EC from 1.48 to 2.90, 0.94 to 1.52, 0.82 to 1.27, and 2.11 to 3.46 dS m^−1^ for straw rice, sawdust, sugar cane, and tree leaves, respectively. The highest EC (3.46 dS m^−1^) was obtained from tree leaves at a pyrolysis temperature of 800 °C, while the lowest EC (0.82 dS m^−1^) was obtained from sugar cane at a pyrolysis temperature of 400 °C. These results agree with those obtained by Shenbagavalli and Mahimairaja^34^, they found that the EC of biochar (paddy straw, maizestover, groundnut shell, coconut shell, coir waste and prosopis wood) ranged from 0.39 to 4.18 dS m^−1^.

The organic matter (OM) increased with increasing pyrolysis temperature significantly. Increasing the pyrolysis temperature from 400 to 800 °C increased the organic matter from 66.0 to 90.5%, 74.8 to 96.7%, 87.8 to 98.1%, and 71.0 to 95.5% for straw rice, sawdust, sugar cane, and tree leaves, respectively. These results agree with those obtained by Wu *et al*^35^. They found the organic matter of biochar prepared from Typha orientalis was increased from 31.63 to 40.54%, when the pyrolysis temperature increasing from 300 to 500 °C. The highest organic matter content (98.1%) was obtained from sugar cane at a pyrolysis temperature of 800 °C, while the lowest organic matter content (66.0%) was obtained from rice straw at a pyrolysis temperature of 400 °C. These results agree with those obtained by^30,36^. They found the highest value of organic matter content was found with the biochar synthesized from sugar cane.

Organic carbon (OC) content significantly increased with increasing pyrolysis temperature. Increasing the pyrolysis temperature from 400 to 800 °C increased the organic carbon content from 38.3 to 52.5%, 43.4 to 56.1%, 50.9 to 56.9%, and 41.2 to 55.4% for straw rice, sawdust, sugar cane, and tree leaves, respectively. These results agree with those obtained by Yargicoglu *et al*^37^. They found that the organic carbon content ranged from 23.5 to 78.1%. The highest organic carbon content (56.9%) was from sugar cane at a pyrolysis temperature of 800 °C, while the lowest organic matter content (38.3%) was obtained from straw rice at a pyrolysis temperature of 400 °C.

The total nitrogen (TN) decreased with significantly increasing pyrolysis temperature. Increasing the pyrolysis temperature from 400 to 800 °C decreased the total nitrogen from 0.9 to 0.4%, 1.4 to 0.5%, 1.9 to 1.3%, and 1.5 to 0.7% for straw rice, sawdust, sugar cane, and tree leaves, respectively. These results agree with those obtained by Jindo *et al*^5^, they found that the total nitrogen decreased from 0.76 to 0.34%, 0.69 to 0.32%, 0.69 to 0.22%, and 1.22 to 0.25% for apple tree, tree oak, rice husk, and rice straw, respectively, when the pyrolysis temperature increased from 400 to 800 °C. The highest TN content (1.9%) was obtained from sugar cane at a pyrolysis temperature of 400 °C, while the lowest TN content (0.4%) was obtained from straw rice at a pyrolysis temperature of 800 °C.

The potassium (K) content significantly increased with increasing pyrolysis temperature. Increasing the pyrolysis temperature from 400 to 800 °C increased the potassium content from 0.6 to 1.6%, 1.3 to 2.7%, 2.2 to 3.5%, and 1.8 to 2.9% for straw rice, sawdust, sugar cane, and tree leaves, respectively. The highest potassium content (3.5%) was obtained from sugar cane at a pyrolysis temperature of 800 °C, while the lowest potassium content (0.6%) was obtained from straw rice at a pyrolysis temperature of 400 °C.

The results indicate that the phosphorus (P) content significantly increased with increasing pyrolysis temperature. Increasing the pyrolysis temperature from 400 to 800 °C increased the phosphorus from 47.6 to 62.3, 65.5 to 121.3, 77.3 to 134.6, and 59.6 to 70.9 mg kg^−1^ for straw rice, sawdust, sugar cane, and tree leaves, respectively. The results also indicate that the highest phosphorus content (134.6 mg kg^−1^) was obtained from sugar cane at a pyrolysis temperature of 800 °C, while the lowest phosphorus content (47.6 mg kg^−1^) was obtained from straw rice at a pyrolysis temperature of 400 °C.

The results also indicate that the calcium (Ca) content significantly increased with increasing pyrolysis temperature. Increasing the pyrolysis temperature from 400 to 800 °C increased the calcium content from 241.3 to 264.2, 491.6 to 546.1, 513.1 to 649.0, and 353.7 to 444.9 mg kg^−1^ for straw rice, sawdust, sugar cane, and tree leaves, respectively. The highest calcium content (649.0 mg kg^−1^) was obtained at from sugar cane at a pyrolysis temperature of 800 °C, while the lowest calcium content (241.3 mg kg^−1^) was obtained from straw rice at a pyrolysis temperature of 400 °C.

The magnesium (Mg) content significantly increased with increasing pyrolysis temperature. Increasing the pyrolysis temperature from 400 to 800 °C increased the magnesium content from 10.9 to 13.2, 21.4 to 27.8, 47.2 to 51.7, and 30.7 to 39.0 mg kg^−1^ for straw rice, sawdust, sugar cane, and tree leaves, respectively. The highest magnesium content (51.7 mg kg^−1^) was obtained from sugar cane at a pyrolysis temperature of 800 °C, while the lowest magnesium content (10.9 mg kg^−1^) was obtained from straw rice at a pyrolysis temperature of 400 °C.

The sodium (Na) content also significantly increased with increasing pyrolysis temperature. Increasing the pyrolysis temperature from 400 to 800 °C increased the sodium content from 1124 to 1329, 1034 to 1109, 1604 to 1703, and 1204 to 1509 mg kg^−1^ for straw rice, sawdust, sugar cane, and tree leaves, respectively. The highest sodium content (1703 mg kg^−1^) was obtained from sugar cane at a pyrolysis temperature of 800 °C, while the lowest sodium content (1124 mg kg^−1^) was obtained from straw rice at a pyrolysis temperature of 400 °C.

The sulfur (So_4_) content increased with increasing pyrolysis temperature. Increasing the pyrolysis temperature from 400 to 800 °C increased the sulfur content from 3568 to 4360, 9752 to 10,138, 11,235 to 12,060, and 10,334 to 11,241 mg kg^−1^ for straw rice, sawdust, sugar cane, and tree leaves, respectively. The highest sulfur content (12,060 mg kg^−1^) was obtained from sugar cane at a pyrolysis temperature of 800 °C, while the lowest sulfur content (3568 mg kg^−1^) was obtained from straw rice at a pyrolysis temperature of 400 °C.

## Conclusions

Yield, physical and chemical properties of biochar synthesized from different agricultural wastes (straw rice, sawdust, sugar cane plant residues, and tree leaves) under different pyrolysis temperature (400, 600, and 800 °C) were determined. The results revealed that yield was affected by both temperature and content of raw materials, where, sugar cane gave the highest yield (450.1 g kg^−1^) compared to other materials. The moisture content of biochar ranged from 1.11 to 4.18%, and the WHC ranged from 12.9 to 27.6 g water g^−1^ dry. The bulk density ranged from 161.5 to 211.9 kg m^−3^. The porosity ranged from 45.9 to 63.7%. The pH ranged from 6.6 to 10.4, and the EC ranged from 0.82 to 3.46 dS m^−1^. The total organic matter content ranged from 66.0 to 98.1%, the total organic carbon content ranged from 38.3 to 56.9%, and the TN content ranged from 0.4 to 1.9%. The total K content ranged from 0.6 to 3.5%. The P and Ca content ranged from 47.6 to 134.6 and 241.3 to 649.0 mg kg^−1^, respectively, for different compost types. The magnesium, sodium, and sulfur content ranged from 10.9 to 51.7, 1124 to 1703, and 3568 to 12,060 mg kg^−1^, respectively. More studies are recommended on biochar properties with mixed biomass. Further studies are recommended to study the feasibility of using biochar in soil improvement compared to other commercial fertilizers.

## Data Availability

The datasets used and/or analyzed during the current study available from the corresponding author on reasonable request.
